# Human papillomavirus vaccine knowledge, health beliefs, recommendation receipt, and intentions among Spanish-speaking Hispanic/Latino sexual and gender minority young adults assigned male at birth in Florida and Puerto Rico: results of a cross-sectional survey

**DOI:** 10.1080/13557858.2025.2607709

**Published:** 2026-01-23

**Authors:** Shannon M. Christy, Steven K. Sutton, Heather Owens, Rolando F. Trejos, Mariana Arevalo, Cathy D. Meade, Jomar Lopez, Lisa J. Sanders, Susan T. Vadaparampil, Erin Park, Melisa Ramos-Sepúlveda, Juliana Borrego-Villanueva, Cyril Patra, Julian Sanchez, Melissa Marzán-Rodríguez

**Affiliations:** a Department of Health Outcomes and Behavior, H.Lee Moffitt Cancer Center and Research Institute, Tampa, Florida, USA; b Morsani College of Medicine, University of South Florida, Tampa, Florida, USA; c Department of Gastrointestinal Oncology, H.Lee Moffitt Cancer Center and Research Institute, Tampa, Florida, USA; d Center for Immunization and Infection Research in Cancer, H.Lee Moffitt Cancer Center and Research Institute, Tampa, Florida, USA; e Department of Biostatistics and Bioinformatics, H.Lee Moffitt Cancer Center and Research Institute, Tampa, Florida, USA; f College of Public Health, University of South Florida, Tampa, Florida, USA; g Independent Researcher; h Alabama College of Osteopathic Medicine, Dothan, Alabama, USA; i Public Health Program, Ponce Health Sciences University, Ponce, PR, USA; j Non-Therapeutic Research Office, H.Lee Moffitt Cancer Center and Research Institute, Tampa, Florida, USA

**Keywords:** Human papillomavirus vaccination, cancer prevention, young adults, sexual and gender minority, gay and bisexual individuals, Hispanic/Latino individuals, Good health and well-being, Reduced inequalities

## Abstract

**Objectives:**

Despite an effective human papillomavirus (HPV) vaccine, uptake among sexual and gender minority (SGM) young adults remains suboptimal, including among Hispanic/Latino SGM. This study aimed to describe awareness/knowledge, health beliefs, attitudes, clinician recommendation receipt, and HPV vaccine intentions among unvaccinated Spanish-speaking Hispanic/Latino SGM young adults assigned male at birth in Florida and Puerto Rico.

**Design:**

Participants completed a cross-sectional online survey between August 2021 and August 2022. Eligibility criteria included being 18–26 years old, male sex assigned at birth, identifying as Hispanic/Latino, identifying as gay, bisexual, or queer, speaking Spanish, living in Florida or Puerto Rico, and having access to the internet. Survey items assessed previous healthcare experiences, HPV vaccine awareness, knowledge, beliefs, attitudes, discussions, clinician recommendation receipt, and vaccine intentions. Descriptive analyses were performed.

**Results:**

Among the 102 unvaccinated participants, all (100%) identified as gay and/or bisexual. Most participants self-reported male gender (96%), being of Puerto Rican descent (92%), and living in Puerto Rico (86%). Only 18.6% of participants reported having received a clinician recommendation for the HPV vaccine. HPV vaccine knowledge was low (Mean=3.2; Standard Deviation [SD] = 2.6; Range: 0–9), attitudes were neutral (Mean=2.5; SD = 0.7; range=1.0–4.3), and perceived barriers were moderate (Mean=2.4; SD = 1.0; range=1.0–4.7). Approximately 35% reported being very likely to seek additional HPV vaccination information in the next year. Approximately one-in-nine (11.8%) reported being very likely to receive the HPV vaccine in the next year, whereas approximately one-third (30.4%) reported being very likely to receive the vaccine at some point in the future.

**Conclusion:**

Findings suggest potential modifiable and multilevel targets for future interventions to promote HPV vaccination among Spanish-speaking Hispanic/Latino SGM young adults assigned male at birth. Such multilevel interventions could address specific knowledge gaps, beliefs, and attitudes among patients and promote clinician recommendations in order to improve HPV vaccination rates among Hispanic/Latino SGM young adults.

## 1. Introduction

Human papillomavirus (HPV) is the most common sexually transmitted infection (STI) worldwide (Bruni et al. 2023), associated with approximately 47,199 new cancer cases in the United States (U.S.) annually (Centers for Disease Control and Prevention 2024a). The burden of HPV is disproportionately experienced by individuals from sexual minority groups, racially and ethnically diverse individuals, and uninsured and underinsured individuals (Amiling et al. 2021; Wheldon, Eaton, and Watson 2023; Meites, Wilkin, and Markowitz 2022; Deshmukh et al. 2023). Compared to heterosexual men, gay, bisexual, and other sexual minority men are 2–6 times more likely to have an HPV infection (Sonawane et al. 2023; Wei et al. 2023). Furthermore, sexual minority men (SMM) have a 17-fold increased risk for developing anal cancer compared to other men (Centers for Disease Control and Prevention 2024c).

Given the absence of universal HPV-related cancer screening guidelines for those assigned male sex at birth (Arnold et al. 2022), HPV vaccination is a crucial method for preventing HPV-related cancers in those assigned male at birth, including sexual and gender minority (SGM) individuals (Chow et al. 2021). Initially, a quadrivalent HPV vaccine was approved for use in those assigned female at birth in 2006, and its use was expanded to include those assigned male at birth in 2009 (American Academy of Pediatrics 2006; Centers for Disease Control and Prevention 2011). The current nonavalent HPV vaccine is approved by the Food and Drug Administration for those aged 9–45 years (National Cancer Institute 2024a), and is recommended for individuals aged 9–26 years (regardless of sex assigned at birth, sexual orientation, or gender identity) (Meites et al. 2019). For those assigned male sex at birth, the nonavalent HPV vaccine can prevent penile, anal, and oropharyngeal cancers and genital warts (Merck 2025). Despite an effective and safe vaccine (Bloem and Ogbuanu 2017), HPV vaccine uptake rates have remained suboptimal in the U.S. and U.S. territories (National Cancer Institute 2024b). Although young adults (regardless of sexual orientation) are at high risk for HPV exposure (Satterwhite et al. 2013), only 39.9% received at least one dose in 2018 (Boersma and Black 2020).

Researchers have applied various health behavior theories to characterize HPV vaccination uptake, including the Theory of Planned Behavior (TPB) and Health Belief Model (HBM) (Chan et al. 2015; Drokow et al. 2021; Gargano et al. 2014; Gerend and Shepherd 2012). Perceived susceptibility, attitudes, subjective norms, and cues to action are associated with HPV vaccine behaviors among young adult SGM (Wheldon, Eaton, and Watson 2023; Gerend et al. 2019). Multiple other factors have been found to influence HPV vaccination behaviors among young adult SGM including personal characteristics (e.g., age, relationship status, racial/ethnic background) (Nadarzynski et al. 2021; Pham et al. 2022), HPV vaccine knowledge (Gerend et al. 2019; Nadarzynski et al. 2021; Nadarzynski et al. 2017), sexual orientation and gender identity (SOGI) disclosure to one’s clinician (Gerend et al. 2019; Nadarzynski et al. 2021), engagement in other health behaviors (e.g., STI screenings) (Nadarzynski et al. 2021; Nadarzynski et al. 2017), and clinician recommendation (Gerend et al. 2019; Pham et al. 2022).

Hispanic/Latino people represent the largest U.S. ethnoracial minority population group, composing approximately 19% of the population (U.S. Census Bureau 2025; Pina and Martinez 2025; Funk and Lopez 2025). Notably, Spanish-speaking Hispanic/Latino people in the U.S. tend to face more healthcare-related discrimination and often encounter additional barriers to accessing preventive care, including receiving provider recommendations for age-appropriate services (Becerra et al. 2014; Escobedo, Cervantes, and Havranek 2023). In the U.S., Hispanic/Latino SMM individuals have consistently had lower HPV and HPV vaccine awareness compared to non-Hispanic White and Black SMM individuals (Chidobem et al. 2022). Furthermore, Hispanic/Latino SMM individuals assigned male at birth have been found to have lower HPV vaccination rates (45.3%) compared to their non-Hispanic White and non-Hispanic Asian/Native Hawaiian/Pacific Islander counterparts (58.3% and 59.8%, respectively) (Amiling et al. 2021).

Although prior studies have sought to examine trends and barriers to HPV vaccination among SGM (Meites, Wilkin, and Markowitz 2022), many studies fail to focus on populations experiencing multiple social identities (e.g., language preference, sexual orientation, sex assigned at birth, gender identity, ethnicity), such as young adult Hispanic/Latino SGM. For example, prior studies have not focused upon the impact of knowledge, health beliefs, and other theory-driven predictors for HPV vaccination outcomes among Hispanic/Latino young adult SGM individuals (Marzán-Rodríguez et al., unpublished data). In addition, Spanish-speaking Hispanic/Latino SGM individuals have been infrequently included in HPV vaccination studies (Marzán-Rodríguez et al., unpublished data). An intersectional perspective that tackles the impact of combinations of social categories can aid in addressing HPV vaccination disparities among young adult SGM individuals, especially those from ethnically-diverse backgrounds (Peterson et al. 2022). The current study was designed to inform a future intervention aimed at promoting HPV vaccination among Spanish-speaking Hispanic/Latino SGM individuals, recognizing that Spanish-speaking Hispanic/Latino SGM individuals may face unique barriers to vaccination. Utilizing a survey informed by the HBM and TPB, the current study aims to describe HPV and HPV vaccination knowledge, awareness, health beliefs, attitudes, healthcare experiences, clinician recommendations, and intentions among unvaccinated Hispanic/Latino Spanish-speaking young adult SGM individuals living in Florida and Puerto Rico.

## 2. Materials and methods

### 2.1. Design

This study is a pilot project conducted in the context of a National Cancer Institute-funded partnership between Ponce Health Sciences University in Puerto Rico and Moffitt Cancer Center in Florida which aimed to address cancer disparities in the catchment areas served by those institutions. Thus, the quantitative study was conducted among individuals living in Florida and Puerto Rico in order to inform future interventions as well as community outreach activities. The study was guided by a Community Advisory Board, which utilized community-engaged approaches to culturally-adapt existing HPV vaccination education materials (a slide presentation that was generally focused on HPV vaccination among adolescents) for Hispanic/Latino SGM individuals assigned male at birth ages 18–26. The current manuscript describes a cross-sectional, Spanish-language online survey which assessed HPV and HPV vaccine knowledge, beliefs, attitudes, intentions, and healthcare experiences. Study procedures were reviewed and approved by the Moffitt Cancer Center Scientific Review Committee and the Institutional Review Board of record (Advarra; PRO00046114).

### 2.2. Recruitment strategies and eligibility

Recruitment strategies included in-person community events in Puerto Rico and Florida (e.g., Pride events, local university and student group events), word of mouth, flyers (e.g., posted at clinics, universities, bars, and restaurants), social media posts, and advertisements on a dating app (i.e., Grindr^®^). In Puerto Rico, additional recruitment was conducted by emailing potentially eligible participants from a previous study who had consented to be contacted. Additionally, a Respondent Driven Sampling approach utilized recruitment seeds to further reach participants; those who successfully referred another individual (max referrals = 3) had the opportunity to receive a referral incentive ($5 gift card). Participants were eligible for the online survey if they self-reported being Spanish-speaking, 18–26 years old, of Hispanic/Latino ethnicity, assigned male sex at birth, having had sex with a man and/or being attracted to men, lived in Puerto Rico or Florida, and had internet access. During the course of the study, the eligibility criteria and survey were amended to allow for those who had received the HPV vaccine to participate.

### 2.3. Survey procedures, challenges, and solutions

The Participant Research, Interventions, and Measurement Core at Moffitt Cancer Center programmed a Spanish-language online survey in Qualtrics (Qualtrics 2024). Following a brief eligibility screener, participants consented to participate in the study by proceeding to the survey after reviewing study information. The survey was designed to be completed in approximately 30 minutes. Participants received a $25 gift card after completing the survey.

The survey was launched in three phases due to fraudulent responses in the initial launch, which required revisions to the survey (e.g., the addition of attention checks). The launch details are as follows. Following initial survey launch (August 2021- mid-October 2021), the study team detected a pattern of suspicious survey submissions suggesting fraudulent participation and/or bots (i.e., software programmed to complete many surveys quickly) (Geers 2024). An example of suspicious submissions includes more than 3 survey submissions from the same IP address. Access to the survey and recruitment efforts were halted. The study team developed a decision tree based on recommendations from published literature to review and remove fraudulent entries based on survey completion length and suggestions of multiple submissions (Bernerth, Aguinis, and Taylor 2021). To prevent and reduce future fraudulent behavior, additional ReCAPTCHA tasks and attention check questions were added to the survey for a second survey launch (March 2022–June 2022; see Supplementary Table S1) (Chandler et al. 2019). Additionally, the study team created QR codes encoding unique survey links to disseminate at recruitment events and via email to prevent duplicate use. In the third launch (July 2022–August 2022), some of the attention checks were removed (see Supplementary Table S1). When possible, a team member emailed potentially fraudulent submissions using the email provided in the survey to request the respondent call and confirm personal information before sending the incentive for survey completion. Submissions deemed fraudulent were excluded.

### 2.4. Measures

The survey assessed multiple sociodemographic, health belief, attitudinal, health care experiences, HPV vaccine discussion, clinician recommendation, and HPV vaccine intention variables (see Supplementary Table S1).

#### 2.4.1. Sociodemographics

Participants were asked to respond to 12 items assessing the following sociodemographic variables: age, sex assigned at birth, race, ethnicity, gender, sexual orientation, primary residence, highest education attained, relationship status, employment status, income, and health insurance status (see Supplementary Table S1).

#### 2.4.2. Health care experiences

Prior health care experiences were assessed using 12 items adapted from existing instruments. Participants were asked if they had a primary source of care and the length of time since their last routine check-up (National Cancer Institute 2024c). Additional vaccine behaviors evaluated were receipt of flu shot in the past 12 months, receipt of tetanus shot in the past 10 years, and receipt of any doses of a COVID-19 vaccine (Centers for Disease Control and Prevention 2024b). Participants were asked if they had a prior diagnosis of HPV, knew someone with an HPV diagnosis, genital or anal warts, or other sexually transmitted infections, and if they received a recommendation for an anal pap test and had received an anal pap test (Reiter et al. 2010; Brewer et al. 2010).

#### 2.4.3. HPV vaccine awareness and knowledge

HPV and HPV vaccine awareness were assessed through two items that asked if they had previously heard of HPV and the HPV vaccine (National Cancer Institute 2024d). HPV and HPV vaccine knowledge were assessed using 19 and 9 items, respectively, adapted from existing measures (Perez et al. 2016; Scherr et al. 2016; Vadaparampil et al. 2016; Kasting et al. 2018). Scores were calculated by summing the number of correct responses.

#### 2.4.4. HPV vaccine beliefs

Six HPV health beliefs were assessed using existing and modified items: perceived risk (Gerend and Shepherd 2012), comparable risk (Lipkus et al. 2000), descriptive norms (Wheldon et al. 2016; Hunt and Gross 2009), perceived norms (Wheldon et al. 2016; Hunt and Gross 2009), perceived barriers (Gerend, Shepherd, and Lustria 2013), and self-efficacy (Champion, Skinner, and Menon 2005). An additional three items modified from existing measures assessed perceived effectiveness of the HPV vaccine in prevention of genital warts, penile cancer, and anal cancer (Dempsey, Fuhrel-Forbis, and Konrath 2014; McRee et al. 2010).

#### 2.4.5. HPV vaccine attitudes

HPV vaccine attitudes were assessed using five items on a 5-point Likert scale modified from existing measures (Dempsey, Fuhrel-Forbis, and Konrath 2014; McRee et al. 2010).

#### 2.4.6. HPV vaccine discussions, clinician recommendation receipt, and HPV vaccine intentions

Participants were asked if they had ever discussed the HPV vaccine with a healthcare provider (i.e., their physician/doctor, a nurse, or another healthcare provider), family (i.e., parent, sibling, or another family member), a friend, significant other/spouse, or ‘I have not discussed the HPV vaccine with any of the above’ (Reiter et al. 2010; Brewer et al. 2010). Clinician recommendation of the HPV vaccine was evaluated through two items modified from existing items that assessed ever receiving a provider recommendation for the HPV vaccine and receiving a provider recommendation for the HPV vaccine in the last 12 months (Rosenthal et al. 2011). Three items were shortened and modified from existing measures assessing HPV vaccine intentions: (1) the likelihood of getting more information about the HPV vaccine in the next year; (2) the likelihood of receiving at least one dose of the HPV vaccine in the next year; and (3) the likelihood of receiving at least one dose of the HPV vaccine at some point in the future (Gerend and Shepherd 2012).

### 2.5. Data review and statistical analyses

Prior to data analysis, participant data from the first launch was evaluated for the date of survey completion (fraudulent completions by bots were evident on a particular date), incomplete survey submissions, the same IP address on three or more surveys, and survey completion time of less than 10 minutes. Surveys meeting any of the above criteria were removed from the dataset. Responses from subsequent launches were evaluated for taking >7 days to complete the survey and providing contradictory answers to survey attention check questions. The quality of open-ended items and instances of straight lining (filling in the same response for all items in a scale) was also assessed; no survey responses were removed with this quality check.

**Figure 1.**
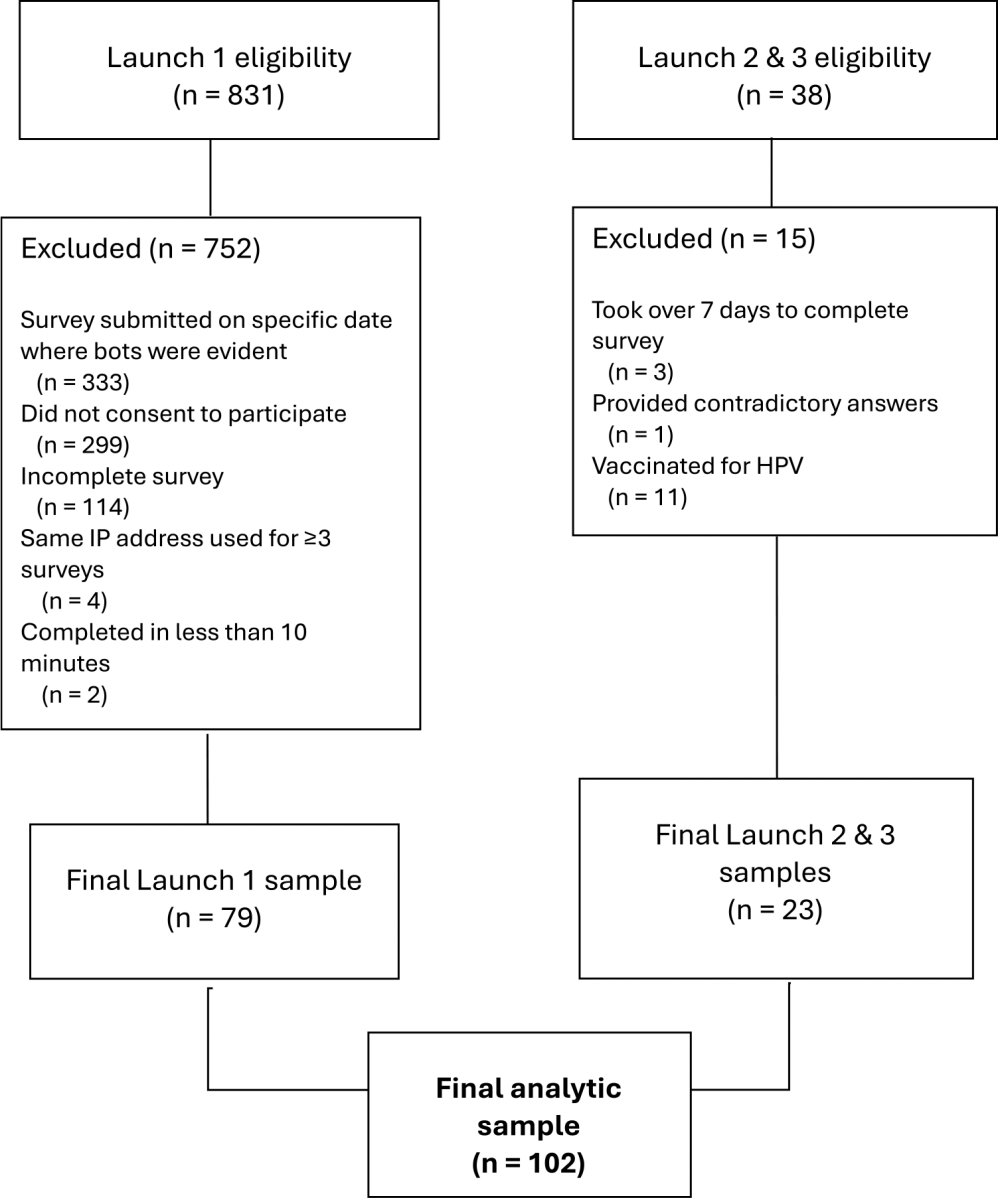
Decision tree for final analytic sample.

Figure 1 shows the number of participants recruited in each launch and number of responses removed in each data quality check. Out of 831 responses in the first survey launch, 752 were excluded for submission on the date the bots were identified, not consenting, incomplete surveys, same IP address used for more than 3 surveys, and survey completion time of less than 10 minutes. In launches 2 and 3, 38 participants completed the survey, and 15 were excluded for taking too long to complete the survey or providing contradictory answers. In total, 87% of responses for all launches were excluded due to quality concerns.

Descriptive analyses (e.g., frequencies, mean, range) were conducted using SAS version 9.4 (SAS Institute Inc., Cary, North Carolina). Spearman correlations examined associations between knowledge, beliefs, attitudes, and the three intentions variables. Although the overall study included both vaccinated and unvaccinated individuals, due to the small sample size, data from 11 respondents who had received the HPV vaccine were not included in the current analyses. Thus, the final analytic sample size was 102 respondents who had not received the HPV vaccine. Among the final analytic sample, the median completion time was 28.9 minutes, with a fastest time of 13.0 minutes and an interquartile range (IQR) of 22.6–42.7 minutes.

## 3. Results

### 3.1. Participant characteristics

Participant characteristics are presented in Table 1. Most participants self-identified as gay (84.3%), reported being of Puerto Rican descent (92.2%), and were living in Puerto Rico (86.3%).

### 3.2. Health care experiences

Participants’ health care experiences are presented in Table 2. The majority (76.5%) reported having health insurance; however, 58.8% indicated they lacked a primary source of healthcare. About one-half (53.9%) reported having had an annual health exam in the past year. Most participants (88.6%) reported having received the COVID-19 vaccine.

### 3.3. HPV vaccine awareness and knowledge

Most participants (79.4%) indicated that they had previously heard about HPV, and slightly more than half (55.9%) reported having heard of the HPV vaccine. The mean HPV knowledge score was 10.6 (standard deviation [SD] = 5.2; range=0–19) and the mean HPV vaccine knowledge score was 3.2 (SD = 2.6; range=0–9). As can be seen in Table 3, the percent correct varied greatly for both HPV (16.7–86.3%) and HPV vaccination (15.7–77.5%) items.

### 3.4. HPV and HPV vaccine beliefs and attitudes

For comparative HPV risk, approximately 40% reported below or much below average risk and approximately 20% reported not knowing their risk. Response frequencies to each perceived risk item are presented in Supplementary Table S2. The mean score for self-efficacy to receive the HPV vaccine was 4.0 (SD = 0.8; range=1–5). The mean total score for number of perceived barriers (range: 1–5) to receiving the HPV vaccine was 2.4 (SD = 1.0; range=1.0–4.7). Most participants reported neutral perceived support followed by strongly agreeing that important figures support their receipt of the HPV vaccine (Supplementary Table S3).

### 3.5. HPV vaccine attitudes

The average score on the attitudes toward the vaccine scale was a 2.5 out of a maximum of 5 (SD = 0.7; range=1.0–4.3).

### 3.6. HPV vaccine discussions and clinician recommendation receipt

About one-half (55.9%) reported not having discussed the HPV vaccine with others. Among those who had had an HPV vaccine discussion, participants most frequently reported discussions with the following individuals: a friend (18.6%), physician (14.7%), a healthcare provider other than a physician or nurse (12.8%), or a parent (11.8%). Approximately 11% indicated discussing the HPV vaccination with a significant other/spouse and about 9% reported having discussed the HPV vaccine with a nurse. Few reported discussing the HPV vaccine with a sibling or a family member other than a parent or sibling (2.9% and 4.9%, respectively). About one-in-five (18.6%) reported ever having received a clinician recommendation for the HPV vaccine. Approximately one-in-twenty (5.9%) indicated receiving a clinician recommendation in the prior year.

### 3.7. HPV vaccine intentions

Approximately 60% of participants reported being very or somewhat likely to try to get more information about the HPV vaccine in the next year. Approximately one-in-eight (11.8%) indicated being very likely and one-third (30.4%) indicated that they were somewhat likely to receive at least one dose of the HPV vaccine in the next year. Conversely, 13.7% indicated that they would be very unlikely to receive at least one dose of the HPV vaccine in the next year. Approximately one-third (30.4%) reported being very likely and one-fourth (23.5%) being somewhat likely to receive at least one dose of the HPV vaccine at some point in the future. Correlations between knowledge, health beliefs, attitudes, and the three intentions variables are presented in Table 4. Supplementary Table S4 presents frequency of each response to all three intention items.

## 4. Discussion

Only one-fifth of participants (all of whom reported being unvaccinated) indicated that they had received a clinician recommendation for HPV vaccination. Prior research exploring clinician discussions and recommendations found that only 14% of providers discuss sexual orientation and HPV vaccination with young adult male patients, and approximately 25% do not discuss either sexual orientation or HPV vaccination (Wheldon et al. 2018). The majority of participants in this study had health insurance, but only slightly more than half had an annual exam in the past year. This is also reflected in the literature, as young adults report only seeing a health care clinician when necessary (i.e., acute visit), and only 71% of individuals 18–34 years old report a wellness visit in the past year compared to 75% of those 35–49 years old (Glauser 2018; National Center for Health Statistics 2025). Young adult patients, including SGM individuals, need to be encouraged to seek annual check-ups, and clinicians should reduce missed opportunities to recommend the HPV vaccine. COVID-19 vaccination among participants was high (89%). However, the role of vaccine requirements, social influence, and COVID-19 vaccine initiatives was not assessed. Of note, Puerto Rico was observed to have high COVID-19 vaccine uptake rates compared to most of the United States (Centers for Disease Control and Prevention 2023).

Consistent with prior literature, awareness and knowledge of HPV were higher than knowledge about the HPV vaccine in the current study (Chidobem et al. 2022). Knowledge gaps reported in prior literature include thinking HPV only impacts those born female, being unaware of the connection between HPV and anal and oropharyngeal cancers, and believing the vaccine is only available for those born female (Gerend et al. 2019). The majority of participants reported intentions to learn more about the HPV vaccine and approximately half of participants reported intending to receive at least one dose in the next year or at some point in the future. Past literature has found that intentions to receive the vaccine among young adult males were higher among those with prior HPV vaccine knowledge (Cooper et al. 2018). Patient education efforts need to be adapted to better take into account Hispanic/Latino SGM young adults’ perspectives so that education is engaging, increases knowledge about HPV-related cancers, and promotes HPV vaccination.

HPV vaccine beliefs and attitudes varied for participants. Comparative risk for HPV was low as most participants believed themselves to be at lower than average risk or did not know their risk for HPV infection, highlighting the need for tailored interventions for SGM to educate individuals about the risk of HPV infection in young adults and SGM population groups. Although 60% of participants did perceive themselves to be at risk for HPV infection, most were not sure of their risk or did not believe they were at risk for developing HPV-related conditions. Prior research shows that perceived risk for anal and oropharyngeal cancer is lower compared to that of genital warts (Reiter et al. 2024). When exploring normative beliefs, prior research shows that SGM individuals believe that important others would be supportive of them being vaccinated for HPV (Gerend et al. 2019); conversely, most participants in the current study responded neutrally to questions about normative beliefs. As HPV vaccine attitudes are associated with HPV vaccination (Wheldon, Eaton, and Watson 2023), targeted interventions are needed to improve HPV vaccine attitudes among SGM young adults.

Study strengths include a focus on HPV vaccination among SGM young adults assigned male sex at birth, a high-risk and understudied population group. Additionally, the study surveyed Spanish-speaking individuals who identify as Hispanic/Latino, allowing for an examination of perspectives often overlooked in HPV vaccination. A literature review from our team (Marzán-Rodríguez et al., unpublished data) suggests that no prior intervention studies have focused solely on HPV vaccination behaviors among Hispanic/Latino SGM young adults. However, prior intervention studies aimed at promoting HPV vaccination have recruited large proportions of SGM individuals reporting Hispanic/Latino ethnicity (Reiter et al. 2023; Fontenot et al. 2020; Gerend et al. 2021). Study limitations include the cross-sectional design, which limits our ability to infer causality, and the use of self-report data, which may be subject to recall bias. The current study focused on Spanish-speaking individuals and those reporting male sex assigned at birth and living in Florida and Puerto Rico, with the majority reporting living in Puerto Rico; therefore may not be generalizable to other populations of Hispanic/Latino SGM young adults elsewhere and individuals who do not speak Spanish. Furthermore, the sample size was relatively small, also limiting generalizability. Considering the impact of intersecting social identities in health research is important, particularly in ensuring that results are relevant to target communities with unique exposures to marginalization and discrimination (Bauer 2014). Our study sample does not allow for disaggregating participant data to understand the simultaneous interplay of multiple social identities (e.g., gender identity, sexual orientation, acculturation, ethnicity, socioeconomic status, language preference). However, our findings can help inform the development of future intervention strategies for similar populations, and we encourage other researchers to continue to explore HPV vaccination in the context of an intersectional framework incorporating intersectional quantitative methodologies, additional sexual minority population groups, and a larger and more geographically representative sample. The data were collected in 2021 and 2022 as the COVID-19 pandemic continued, which may have impacted individuals’ intentions to receive the HPV vaccine and general vaccine attitudes. Finally, the current study did not examine associations between cancer fatalism (the extent to which one believes that a cancer diagnosis will result in death from that cancer) and HPV vaccine attitudes, beliefs, and intentions. However, a prior study conducted among Hispanic/Latino young adult women found that HPV vaccine uptake was inversely associated with cancer fatalistic beliefs (Taskin et al. 2024). Future research should examine the role of cancer fatalism in HPV vaccination among Hispanic/Latino SGM young adults assigned male at birth. Future studies should replicate and extend the current study to address study limitations and include English- and Spanish-speaking Hispanic/Latino SGM young adults to develop salient patient education messaging in their preferred languages. The long-term goal is the development of multilevel interventions targeted for Hispanic/Latino SGM.

## 5. Conclusion

The current study assessed knowledge, health beliefs, attitudes, and health care experiences among Hispanic/Latino SGM young adults assigned male at birth in Florida and Puerto Rico, revealing that clinician recommendation receipt was low, as were intentions to receive the HPV vaccination in the next year. Findings revealed knowledge gaps as well as perceived barriers and attitudes toward HPV vaccination, which could be addressed in future interventions. Additional research guided by an intersectional perspective is needed to better understand the barriers and facilitators for HPV vaccination among this population. Also, multilevel interventions which promote clinician recommendations and patient education should be developed and tested to improve HPV vaccination rates among this understudied population.

## Supplementary Material

Supp 1

Supplemental data for this article can be accessed online at https://doi.org/10.1080/13557858.2025.2607709.

## Figures and Tables

**Table 1.** Participant characteristics (*N* = 102).

Variable	Frequency	Percent

Age (mean)	23.2 (SD: 2.6)	Range: 18–26
Gender		
Male	98	96.1
Non-Binary	4	3.9
Sexual orientation		
Gay	76	74.5
Bisexual	21	20.6
Both gay and bisexual	5	4.9
None of the above	0	0
Has had sex with a man		
Yes	96	94.1
No, but attracted to men	6	5.9
Prefers information in Spanish		
Yes	79	77.5
No	23	22.6
Primary residence location		
Florida	14	13.7
Puerto Rico	88	86.3
Hispanic, Latino, or Spanish origin		
Mexican American	1	1.0
Puerto Rican	92	90.2
Cuban	4	3.9
Other Hispanic or Latino or Spanish origin	3	2.9
More than one	2	2.0
Race		
White	45	44.1
Black/African American	22	21.6
Other/None of these	23	23.6
More than one	12	11.8
Relationship status		
Married/Living as married	23	22.6
Separated	3	2.9
Dating for longer than 1 week	29	28.4
Dating, but not exclusively	15	14.7
Not dating/married	32	31.4
Annual income		
$0 to $9,999	21	20.6
$10,000 to $14,999	8	7.8
$15,000 to $19,999	13	12.8
$20,000 to $34,999	12	11.8
$35,000 to $49,999	12	11.8
$50,000 to $74,999	19	18.6
$75,000 to $99,999	3	2.9
$100,000 or more	3	2.9
Prefer not to answer	11	10.8
Educational attainment		
Never attended school	1	1.0
Some high school	7	6.9
High school graduate	11	10.8
Some college or technical school	24	23.5
College graduate	46	45.1
Graduate or professional school after college	13	12.8
Employment status		
Employed	53	52.0
Unemployed	11	10.8
Student	35	34.3
Other	3	2.9
Has health insurance		
Yes	78	76.5
No	24	23.5

Notes: SD: standard deviation; Totals may not equal 100% due to rounding.

**Table 2.** Participant health care experiences (*N* = 102).

Variable	Frequency	Percent

Have primary source of care		
Yes	42	41.2
No	60	58.8
Time since last routine check-up		
Within past year (anytime less than 12 months ago)	55	53.9
More than 1 year but less than 2 years ago	22	21.6
More than 2 years but less than 5 years ago	8	7.8
5 or more years ago	8	7.8
I don’t know	6	5.9
Never	3	2.9
Diagnosed with HPV		
Yes	4	3.9
No	90	88.2
I’m not sure	8	7.8
Knows someone who has been diagnosed with HPV		
Yes	82	80.4
No	12	11.8
I’m not sure	8	7.8
Ever had genital warts		
Yes	6	5.9
No	88	86.3
I’m not sure	8	7.8
Ever had anal warts		
Yes	4	3.9
No	89	87.3
I’m not sure	9	8.8
Ever diagnosed with a sexually transmitted infection		
Yes	13	12.8
No	85	83.3
I’m not sure	2	2.0
I prefer not to answer	2	2.0
Ever been recommended to receive an anal pap test		
Yes	5	4.9
No	93	91.2
I don’t know	4	3.9
Ever received an anal pap test		
Yes	6	5.9
No	93	91.2
I’m not sure	3	2.9
Flu shot in past 12 months		
Yes	27	26.5
No	61	59.8
I’m not sure	14	13.7
Tetanus vaccine in past 10 years		
Yes	45	44.1
No	36	35.3
I’m not sure	21	20.6
Received any COVID-19 vaccine doses		
Yes	70	88.6
No	9	11.4
Missing	23	–

Notes: HPV: human papillomavirus; COVID-19: coronavirus disease; Totals may not equal 100% due to rounding.

**Table 3.** Human papillomavirus and human papillomavirus vaccine knowledge responses by item.

Item	True n(%)	False n(%)	Do not know n(%)

**HPV Knowledge**			
HPV is very rare	17 (16.7)	55 (53.9)	30 (29.4)
HPV always have visible signs or symptoms	29 (28.4)	43 (42.2)	30 (29.4)
HPV can cause cervical cancer	67 (65.7)	1 (1.0)	34 (33.3)
There are many types of HPV	51 (50.0)	4 (3.9)	47 (46.1)
HPV can cause HIV/AIDS	19 (18.6)	52 (51.0)	31 (30.4)
HPV can be transmitted through vaginal sex	71 (69.6)	3 (2.9)	28 (27.5)
HPV can cause genital warts	70 (68.6)	0 (0)	32 (31.4)
Men cannot get HPV	5 (4.9)	82 (80.4)	15 (14.7)
Using condoms reduces the chances of HPV transmission	88 (86.3)	4 (3.9)	10 (9.8)
HPV can be cured with antibiotics	8 (7.8)	46 (45.1)	48 (47.1)
Most sexually active people will get HPV at some point in their lives	45 (44.1)	13 (12.8)	44 (43.1)
A person could have HPV for many years without knowing it	78 (76.5)	0 (0)	24 (23.5)
HPV can cause anal cancer	47 (46.1)	5 (4.9)	50 (49.0)
HPV can be transmitted through oral sex	65 (63.7)	3 (2.9)	34 (33.3)
HPV can cause cancer of the penis	36 (35.3)	6 (5.9)	60 (58.8)
HPV can be transmitted through anal sex	74 (72.6)	0 (0)	28 (27.5)
HPV infections always lead to health problems	51 (50.0)	17 (16.7)	34 (33.3)
HPV can cause oral cancer	40 (39.2)	2 (2.0)	60 (58.8)
A person with no symptoms cannot transmit the HPV infection	15 (14.7)	56 (54.9)	31 (30.4)
**HPV Vaccine Knowledge**			
The HPV vaccine requires at least 2 doses	28 (27.5)	4 (3.9)	70 (68.6)
The HPV vaccine offers protection against all sexually transmitted infections	3 (2.9)	79 (77.5)	20 (19.6)
The HPV vaccine is most effective when given to people who have never had sex	26 (25.5)	26 (25.5)	50 (49.0)
The HPV vaccine offers protection against most cervical cancers	19 (18.6)	18 (17.7)	65 (63.7)
The HPV vaccine offers protection against genital warts	32 (31.4)	11 (10.8)	59 (57.8)
The HPV vaccine protects you from all types of HPV	29 (28.4)	16 (15.7)	57 (55.9)
You can cure HPV by getting the HPV vaccine	13 (12.8)	34 (33.3)	55 (53.9)
Males and females can receive the HPV vaccine starting at age 9	40 (39.2)	11 (10.8)	51 (50.0)
Both males and females can receive the HPV vaccine between the ages of 9–45	52 (51.0)	3 (2.9)	47 (46.1)

Notes. HIV: Human Immunodeficiency Virus; AIDS: Acquired Immunodeficiency Syndrome; Values in each cell are n (%). Row totals may not equal 100% due to rounding. Underlining denotes the correct response.

**Table 4.** Correlations between select study variables.

	Likelihood of getting more information about the HPV vaccine in the next year (r)	Likelihood of getting at least one dose of HPV vaccine in the next year (r)	Likelihood of getting at least one dose of HPV vaccine at some point in the future (r)

HPV Awareness	0.23	0.25	0.23
HPV Vaccine Awareness	0.10	0.15	0.13
Perceived Barriers	−0.06	−0.19	−0.26
Self-efficacy	0.036	0.18	0.19
Vaccine Attitudes	0.10	0.20	0.19
HPV Knowledge Score	0.12	0.10	0.15
HPV Vaccination Knowledge Score	0.10	0.07	0.10

Notes. HPV: human papillomavirus; r denotes Spearman’s correlation.

## Data Availability

De-identified data will become available by the corresponding author upon reasonable request.
