# Supplementary material for: Human papillomavirus vaccine knowledge, health beliefs, recommendation receipt, and intentions among Spanish-speaking Hispanic/Latino sexual and gender minority young adults assigned male at birth in Florida and Puerto Rico: results of a cross-sectional survey
Source: Ethn Health. Author manuscript; Available in PMC 2026 Apr 4. (PMC13048128; doi:10.1080/13557858.2025.2607709)
Supplement: Supp 1 [file NIHMS2150130-supplement-Supp_1.docx]

**Table S1. Variables Utilized across Three Survey Launches**

| **Variable** | **Number of Items** | **Launch 1** | **Launch 2** | **Launch 3** | **Notes** |
| --- | --- | --- | --- | --- | --- |
| Sex assigned at birth | 1 | X | X | X | Launches 2 & 3 included Intersex |
| Hispanic, Latino, or Spanish Ethnicity | 1 | X | X | X |  |
| Read and write Spanish | 1 | X | X | X |  |
| Prefer to receive medical information in Spanish | 1 | X | X | X |  |
| HPV vaccination status | 1 | X |  |  | Used to determine eligibility in Launch 1 |
| Residence | 1 | X | X | X |  |
| Have you engaged in oral or anal sex with another man | 1 | X | X | X | Individuals could indicate “No, but I am attracted to men” and be eligible |
| Access to internet | 1 | X | X | X |  |
| Health care utilization | 3 | X | X | X |  |
| Primary care provider knows sexual orientation | 2 | X | X | X | Launches 2 & 3 include ‘them’ in “I [do/don’t] talk to him/her/them regarding my sexual orientation” |
| HPV and HPV vaccine awareness | 2 | X | X | X |  |
| HPV knowledge | 19 | X | X | X | Cronbach’s alpha=0.89 |
| HPV vaccine knowledge | 9 | X | X | X | Cronbach’s alpha=0.82 |
| Discussions about vaccine | 1 | X | X | X | Launches 2/3 added ‘please specify’ |
| HPV vaccine recommendation | 4 | X | X | X |  |
| Vaccination status | 1 |  | X | X |  |
| Number of doses received | 1 |  | X | X |  |
| Age vaccinated | 1 |  | X | X |  |
| Perceived risk | 5 | X | X | X | Cronbach’s alpha=0.97.  Launch 1 included “I don’t know” |
| Comparable risk | 1 | X | X | X |  |

| **Variable** | **Number of Items** | **Launch 1** | **Launch 2** | **Launch 3** | **Notes** |
| --- | --- | --- | --- | --- | --- |
| Perceived norms | 8 | X | X | X | Cronbach’s alpha=0.95.  Launches 2 & 3 included “Not applicable” |
| Perceived barriers | 3 | X | X | X | Cronbach’s alpha=0.66.  Launch 1: not at all, a little, somewhat, a lot, a great deal. Launches 2 & 3: not at all concerned, a little concerned, somewhat concerned, very concerned |
| Self-efficacy | 9 | X | X | X | Cronbach’s alpha=0.87 |
| Intention | 3 | X | X | X |  |
| Vaccine attitudes and beliefs | 8 | X | X | X | Cronbach’s alpha=0.63 |
| Education preferences | 3 | X | X | X |  |
| Vaccine preferences | 1 | X | X | X |  |
| Sexual health comfort | 3 | X | X | X |  |
| Health history | 6 | X | X | X | HIV question clarifies that “HPV is not HIV or herpes.” in launches 2 & 3 |
| Anal pap smear | 3 | X | X | X |  |
| Prior flu vaccination | 1 | X | X | X |  |
| Prior tetanus vaccination | 1 | X | X | X |  |
| Prior COVID vaccination | 1 | X | X | X | Launch 1 asks this question after COVID-19 impact section |
| Sexual experiences | 9 | X | X | X |  |
| COVID-19 impacted healthcare use | 1 | X | X | X | Launch 1: “Has the COVID-19 pandemic impacted you going to see your primary care or family medicine provider”. Launches 2 & 3: “Has the COVID-19 pandemic impact your health care use?” – Yes –“How has COVID-19 impacted your healthcare use?” |
| Delay in care due to COVID-19 | 1 | X | X | X |  |

| **Variable** | **Number of Items** | **Launch 1** | **Launch 2** | **Launch 3** | **Notes** |
| --- | --- | --- | --- | --- | --- |
| COVID-19 vaccine intentions | 2 | X |  |  |  |
| Race | 1 | X | X | X | Launches 2 & 3 ‘None of these/Other’ asks to please specify |
| Education | 1 | X | X | X |  |
| Relationship Status | 2 | X | X | X |  |
| Sexual Orientation | 1 | X | X | X | Launches 2 & 3 ‘Not listed above’ asked to please write in |
| Gender identity | 1 | X | X | X |  |
| Employment status | 1 | X | X | X | Launches 2 & 3 ‘other’ asked to please specify |
| Income | 1 | X | X | X |  |
| Health insurance status | 1 | X | X | X |  |
| Type of health insurance | 1 | X | X | X | Launches 2 & 3 provide additional examples of private insurance |
| Policy holder | 1 | X | X | X | Launches 2 & 3 ‘other’ asked to please specify |
| Age | 1 |  | X | X |  |
| Sex assigned at birth | 1 |  | X | X |  |
| Ethnicity | 1 |  | X | X |  |
| Primary residence | 1 |  | X | X |  |
| Hispanic/Latino-specific cultural items | 10 | X | X | X |  |
| Venue attendance | 3 |  | X | X |  |

**Note.** HPV: human papillomavirus; HIV: human immunodeficiency virus; COVID-19: coronavirus disease

**Table S2. Perceived Risk Beliefs by Item**

| **Item** | **No chance** | **Very Unlikely** | | **Somewhat Unlikely** | | **Neither likely nor unlikely** | **Somewhat likely** | | **Very likely** | | **Certain I will get** | **I don’t know** | |  |
| --- | --- | --- | --- | --- | --- | --- | --- | --- | --- | --- | --- | --- | --- | --- |
| How likely do you think you are to get HPV in the future? | 8 (7.8) | 4 (3.9) | 3 (2.9) | | 19 (18.6) | | 18 (17.7) | 27 (26.5) | | 16 (15.7) | | | 7 (6.9) | |
| How likely do you think you are to get genital warts in the future? | 17 (16.7) | 17 (16.7) | 9 (8.8) | | 24 (23.5) | | 6 (5.9) | 3 (2.9) | | 2 (2.0) | | | 24 (23.5) | |
| How likely do you think you are to get anal cancer in the future? | 16 (15.7) | 15 (14.7) | 5 (4.9) | | 28 (27.5) | | 4 (3.9) | 1 (1.0) | | 1 (1.0) | | | 32 (31.4) | |
| How likely do you think you are to get oral cancer in the future? | 18 (17.7) | 16 (15.7) | 6 (5.9) | | 23 (22.6) | | 6 (5.9) | 0 (0.0) | | 1 (1.0) | | | 32 (31.4) | |
| How likely do you think you are to get penile cancer in the future? | 20 (19.6) | 14 (13.7) | 6 (5.9) | | 23 (22.6) | | 2 (2.0) | 0 (0.0) | | 1 (1.0) | | | 36 (35.3) | |

***Notes.*** Values in each cell are n (%). Totals may not equal 100% due to rounding.

**Table S3.** **Normative Beliefs by Item**

| **Item** | **Strongly disagree** | **Somewhat disagree** | **Neutral** | **Somewhat agree** | **Strongly agree** | **Not applicable** |
| --- | --- | --- | --- | --- | --- | --- |
| Most people who are important to me would want me to get the HPV vaccine. | 10 (9.8) | 2 (2.0) | 54 (52.9) | 10 (9.8) | 21 (20.6) | 5 (4.9) |
| Most people in my family would want me to get the HPV vaccine. | 9 (8.8) | 7 (6.9) | 57 (55.9) | 6 (5.9) | 17 (16.7) | 6 (5.9) |
| My mother would want me to get the HPV vaccine. | 8 (7.8) | 5 (4.9) | 52 (51.0) | 10 (9.8) | 21 (20.6) | 6 (5.9) |
| My father would want me to get the HPV vaccine. | 8 (7.8) | 6 (5.9) | 59 (57.8) | 4 (3.9) | 18 (17.7) | 7 (6.9) |
| My gay friends would want me to get the HPV vaccine. | 7 (6.9) | 5 (4.9) | 47 (46.1) | 19 (18.6) | 19 (18.6) | 5 (4.9) |
| My straight friends would want me to get the HPV vaccine. | 8 (7.8) | 3 (2.9) | 55 (53.9) | 12 (11.8) | 18 (17.7) | 6 (5.9) |
| My healthcare provider would want me to get the HPV vaccine. | 7 (6.9) | 1 (1.0) | 47 (46.1) | 12 (11.8) | 28 (27.5) | 7 (6.9) |
| My current and/or future sexual partner(s) would want me to get the HPV vaccine. | 7 (6.9) | 2 (2.0) | 43 (42.2) | 16 (15.7) | 28 (27.5) | 6 (5.9) |

***Notes.*** Values in each cell are n (%). Totals may not equal 100% due to rounding.

**Table S4.** **Human Papillomavirus Vaccination Intentions by Item**

| **Item** | **Very**  **unlikely** | **Somewhat unlikely** | **A little unlikely** | **Neither unlikely nor likely** | **A little likely** | **Somewhat likely** | **Very likely** |
| --- | --- | --- | --- | --- | --- | --- | --- |
| How likely is it that you'll try to get more information about the HPV vaccine in the next year? | 15 (14.7) | 2 (2.0) | 5 (4.9) | 13 (12.8) | 5 (4.9) | 26 (25.5) | 36 (35.3) |
| How likely is it that you'll get at least one dose of HPV vaccine in the next year? | 14 (13.7) | 5 (4.9) | 5 (4.9) | 27 (26.5) | 8 (7.8) | 31 (30.4) | 12 (11.8) |
| How likely is it that you’ll get at least one dose of HPV vaccine at some point in the future? | 12 (11.8) | 9 (8.8) | 4 (3.9) | 20 (19.6) | 2 (2.0) | 24 (23.5) | 31 (30.4) |

***Notes.*** Values in each cell are n (%). Totals may not equal 100% due to rounding.
